# Simple, sensitive and robust chicken specific sexing assays, compliant with large scale analysis

**DOI:** 10.1371/journal.pone.0213033

**Published:** 2019-03-01

**Authors:** Liyan He, Priscila Martins, Joris Huguenin, Thi-Nhu-Ngoc Van, Taciana Manso, Therese Galindo, Flavien Gregoire, Lise Catherinot, Franck Molina, Julien Espeut

**Affiliations:** 1 Sys2diag, UMR9005 CNRS/Alcediag, Montpellier, France; 2 Tronico, Saint-Philbert-de-Bouaine, France; University of Helsinki, FINLAND

## Abstract

Chicken meat and eggs are important sources of food for the world population. The significant increase in food demand has pushed the food industry toward a rapid non-expensive production which in turn raises ethical issues. How chicken are cultivated and processed in food industry is no longer acceptable. Ethical and economical concerns emerging from chicken culling need to be solved in the near future. Indeed, in egg production industry, male chicken are killed at the age of 1-day post-hatching since they are not egg producers. A number of laboratory all over the world are looking for innovative non-invasive sexing methods to determine the sex of chicken in the early stages of the development before hatching. It will allow males’ chicken elimination before the pain-feeling stages. In order to evaluate the efficiency of these methods, the scientific community need a reliable, easy to use and cost-effective *in-ovo* invasive sexing method. In this report, we developed two new invasive assays based on PCR and Q-PCR techniques respectively, which fulfil the above mentioned requirements. In the same line with other groups, we exploited the differences betweed males (ZZ) and females (ZW) chicken sexual chromosomes. We identified two genes, SWIM and Xho-I, on chromosome W and DMRT gene on chromosome Z allowing a clear discrimination between the two sexes using PCR and qPCR respectively. These two new genomic markers and their corresponding methods not only increase the accuracy but also reduce time and cost of the test compared to previously developed sexing methods. Depending on the technology available in the lab, one can choose between the two techniques requiring different machines and expertise.

## Introduction

The chicken embryo is a well-established model in developmental biology studies since it allows direct manipulation of living embryo. Moreover, chicken meat and eggs are important sources of food for the global population. 118 million tons of meat and 1400 billion eggs were produced in 2017 (Source: Poultry Trends). In chicken eggs production industry, all males’ chicks are killed at the age of 1 day post-hatching, since only females are valuable. Each year, all over the world, around 6 billion male chicks are considered as useless products (Source: Food and Agriculture Organization of the United Nations). It raises ethical and economical concerns that must be fixed as soon as possible, mostly to avoid animal suffering. Many countries and companies around the world are running scientific programs in order to be able to determine the sex of living chicken embryos at the early stages of development. Once working, it will allow eliminating eggs with male embryos before pain feeling stages (there is a current consensus before day 11 of egg development). Up to date, various assays have been established to sex chicken embryos: specific DNA amplification by PCR or Q-PCR [1–11]; hormones detection [12]; infrared, fluorescence or Raman spectroscopy detection of sex specific signals following egg opening [13–15]; exalted odour analysis by gas chromatography coupled with mass spectrometry [16]. Among these approaches, odour analysis represents the sole non-invasive technique that does not require eggshell opening. However, up to now, this is not yet a reliable method for egg sexing. Other approaches raise a biological concern since egg sexing must be run under non-sterile environment i.e. hatcheries.

A reference *in-ovo* sexing method that is highly reliable, rapid, user friendly and cost effective to validate *ex-ovo* approaches is required for the development of non-invasive assays. In other words, the reference assay serves as a standard to validate future new non-invasive egg sexing assays. PCR is a molecular method that is typically used in chicken sex identification. These assays exploit the genetic differences between males that have two Z sexual chromosomes (ZZ) and females have one Z and one W sexual chromosome (ZW) using different genes as markers. For instance, the Chromo-Helicase-DNA-binding 1 (CHD-1) genes have been commonly used as sex determinants for chicken [6, 7]. CHD-1 genes are located on both Z (CHD-Z) and W (CHD-W) chromosomes in which CHD-Z and CHD-W share common sequences whilst, at the same time, reserve sexual specific sequences allowing sex differentiation with only one pair of primers. Xho-I and EcoR-I (EE0.6) repetitive regions present on W chromosome are also among those that have been used as avian female-specific PCR probes [4, 10]. Nevertheless, none of the previously developed methods was neither assayed in terms of performances nor fully satisfied all requirements of a reference assay such as sensitivity, specificity, robustness, rapidity and low-cost. In order to establish such an assay, we addressed the performances of existing methods and, at the same time, developed two new assays based on PCR and real-time quantitative PCR (Q-PCR) respectively. Both new assays performances were extensively evaluated on 176 chicken embryo samples.

## Materials and methods

### Eggs incubation

Fertilized eggs from 4 different races; ISA Brown, Dekalb White, Bovan Brown and Shaver Black were obtained from a commercial supplier (SFPA, Saint-Marcellin France). Eggs were stocked at 19°C for a maximum of 10 days after laying. Fertile eggs were incubated in a dedicated incubator (Masson, Soyans France) at 37.5 °C, 55% humidity and tilted every hour for 9 days.

### Samples collection and lysis

10–20 mg of day 9 post-hatching embryo brain tissue was pipetted and lysed in 150 μl of lysis buffer containing 10% of chelating beads (Chelex 100 Biorad), 0.2% SDS, 10mM Tris-HCl pH 8, 0.2 μg/μl Proteinase K. Brain was chosen for DNA extraction since it is a soft tissue easy to aspirate with a P1000 pipette. Tissues were incubated for 3h at 55°C following by 15 minute incubation at 95°C. Samples were then centrifuged for 5 minutes at 13000g at room temperature. Supernatant were recovered and stored at -20°C until used. For sensitivity assessment, DNA lysate quantification was performed with Qubit Fluorometer (dsDNA HS (high sensitivity) Assay Kit, Invitrogen) and by reading the 260 nm absorbance with a micro volume spectrophotometer (Nanodrop One Thermo Scientific, Wilmington USA) to estimate the purity of the sample.

### PCR analysis

**(**dx.doi.org/10.17504/protocols.io.uf6etre**)**

Embryo lysates were diluted 10 times in nuclease free water and 1 μl of dilution was mixed on ice with: 9.5 μl nuclease free water, 12.5 μl of 2X Master Mix (Invitrogen Platinum Green Hot Start PCR), 200 nM of primer SWIM and 12S (Table 1). Amplification was performed by using a peqSTAR 96X thermocycler (Ozyme, Montigny-le-Bretonneux France). Thermal cycling conditions for DNA amplification were: 1 cycle of initial denaturation at 94°C for 2 minutes; 35 cycles comprising 30s at 94°C for the denaturation, 30s at 55°C for annealing, 30s at 72°C for the elongation; and a final extension cycle at 72°C for 5 minutes.

**Table 1:** List of PCR and Q-PCR primers used in this study.

Primer name	Targeted gene	Sequence	Specificity	Amplicon size	Reference
**SWIM –F**	SWIM	GAGATCACGAACTCAACCAG	Female	212 bp	Original
**SWIM –R**		CCAGACCTAATACGGTTTTACAG			
**12S –F**	12S r-RNA gene	CTATAATCGATAATCCACGATTCA	Female and Male	131 bp	[8]
**12S –R**		CTTGACCTGTCTTATTAGCGAGG			
**XhoI –F**	Xho-I repeats	CCCAAATATAACACGCTTCACT	Female	415 bp	[19]
**XhoI –R**		GAAATGAATTATTTTCTGGCGAC			
**D57 –F**	DMRT	CTTTCACAAATGTGTTCTGCTGT	Female and Male	57 bp	Original
**D57 –R**		AGCAGATACAACCTAAGAATGCC			

Two different analyses of PCR products were performed: Agarose gel and capillary electrophoresis

- 15 μl of the PCR reactions were loaded on a 2% Gelgreen (Biotium, California USA) stained agarose gel in 0.5X TAE buffer and separated by electrophoresis at 100 Volts. 1 μg of DNA ladder (GeneRuler 50bp, Fisher Scientific, Illkirch France) serves as a reference for the migration. Gels were revealed by Blue LED GelPicBox at 430nm (Nippongenetics, Düren Germany).
- 10 μl of the PCR reactions were analysed by microfluidic capillary electrophoresis systems (Capillary LifeSciences, France) controlled by the Labchip GX version 4.1.1619.0 SP1 software. Capillary electrophoresis is the high throughput format of the traditional gel electrophoresis. These assays allow a rapid size-based separation and sensitive detection of specific DNA fragments via UV absorption or fluorescent labelling [17, 18]. The CE system is more advantageous over the conventional slab gel electrophoresis in terms of speed, high-throughput applicability, automated workflow, resolution, and sensitivity.

### Real-time quantitative PCR (Q-PCR) analysis

**(**dx.doi.org/10.17504/protocols.io.ugaetse**)**

Q- PCR reactions were prepared in 384 multi-well plates. Each 10 μl reaction volume contained: 5 μl of LightCycler 480 SYBR Green I Master Mix (Roche, Meylan France), 1 μl of brain lysate diluted 4 times, 500 nM of each of the 4 primers (Xho-I and D57, Table 1) and 2 μl of nuclease free water. Real-time Q-PCR thermal cycling reactions were performed by the LightCycler 480 (Roche, Meylan France) directed by the LightCycler 480 Software (version 1.5.1.62). Thermal cycling conditions were: Pre-Incubation 5 minutes at 95°C, 45 cycles of DNA amplification—(95°C for 10s, 53°C for 20s, 72°C for 30s). For melting curve analysis, we set up 1 cycle at 95°C for 5s, 65°C for 1 minute, 97°C in continuous mode and 1 cooling cycle at 40°C for 10s.

### Robustness assessment of PCR and Q-PCR sexing assays

176 ISA-brown egg embryo tissues at day 9 of incubation were lysed for PCR and Q-PCR analysis. Lysates were not neither purified nor quantified in order to assess the robustness of the PCR and Q-PCR assays on heterogeneous samples in terms of DNA concentration and contaminants. As reference, we used previously described primers CHD 2250F/2718R[6] for sex determination. All samples were tested separately using our new PCR (with the primers SWIM/12S) and Q-PCR (with the primers Xho-I and D57) protocols.

PCR samples were analysed by capillary electrophoresis (LabChip GX) and the data were processed by an R studio program (Version 0.99.903) that we developed for automatization. Each electropherogram profile was normalized in time with two markers and the baseline was subtracted.

For Q-PCR analysis, Crossing Point (CP) and Melting Curve (MC) analysis were combined to discriminate between females and males. We programed a script in R studio for the routine analysis of the curves.

## Results and discussion

### A new simple and robust PCR assay for chicken sexing

**Fig 1:**
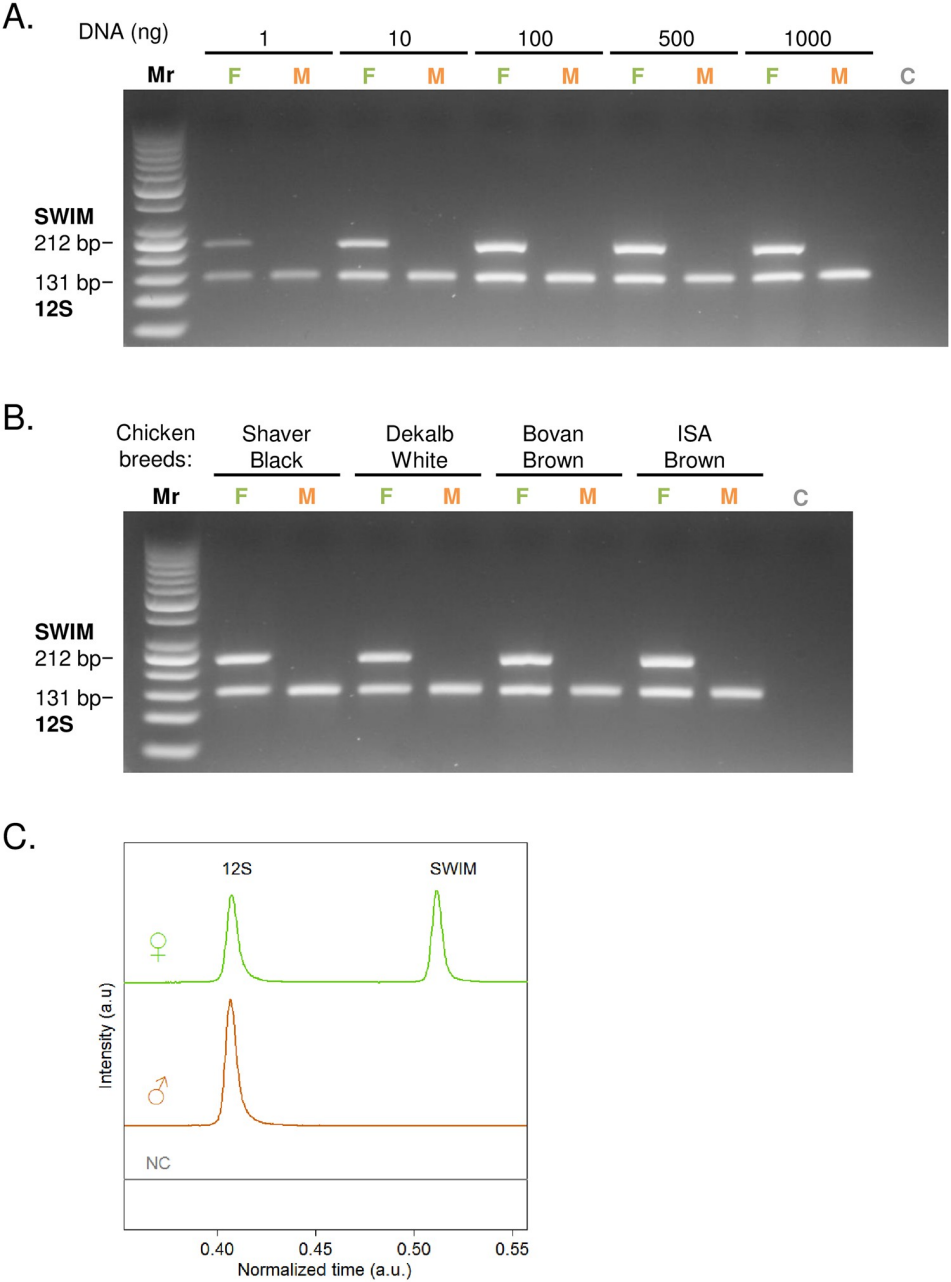
Specificity and sensitivity of the new PCR sexing assay based on SWIM and 12S gene amplification. (**A**) Different crude embryo extracts containing 1 ng to 1000 ng of DNA were tested. Female chicken are detected by the amplification of two amplicons, the SWIM female specific amplicon of 212 bp and the 12S reference amplicon of 131 bp. (**B**) SWIM/12S PCR amplification of 4 sexed chicken breeds; Shaver black, Dekalb white, Isa brown, Bovan brown; on embryo extracts containing 100 ng DNA (M: Male, F: Female, C: Negative control, Mr: 50 bp ladder markers). (**C**) Three typical fluorescence profiles from female (♀), male (♂) and negative control containing no DNA template (NC) obtained by microfluidic capillary electrophoresis analysis. The female samples generated two peaks (SWIM: 212 bp and 12S: 131 bp), while the male samples gave only one peak at 131 bp (12S). No significant signal was observed for the negative control.

In order to find a user friendly and robust PCR egg sexing assay that can be used in high-throughput format, we designed and characterized 64 different DNA primers targeting different *Gallus gallus* genes (S1, S2, S3 and S4 Figs, S1 Table). Among them the coupling of 4 primers amplifying parts of SWIM and 12S genes led to a very specific and sensitive assay (Fig 1). SWIM gene coding for Zinc Finger SWIM domain-containing protein 6 like, is located on W-chromosome therefore serves as specific marker for female. 12S gene coding for mitochondrial ribosomal small subunit is common for males and females thus serves as the reference of the assay. To validate the specificity and sensitivity of the PCR reaction combining SWIM (Female) and 12S (Reference) gene primers, we first evaluated PCR amplification for 5 different DNA concentrations from crude embryo extracts varying from 1 ng to 1 μg (Fig 1A). Sensitivity of the assay was also evaluated using samples from 4 different chicken races (Fig 1B). The experimental procedure is detailed in the materials and methods section.

Our results clearly show that the SWIM/12S PCR amplification is highly specific since there is no miss-amplification even in the presence of large amount of DNA input (1 μg) and no amplification occurring in the absence of DNA template (negative control). SWIM primers amplify a 212 bp band for all DNA extracts from female, while the 12S primers amplify a 131 bp product for both males and females. By capillary electrophoresis analysis, which is more sensitive than agarose gels (Detection of SWIM/12S amplicons from crude extracts containing 0.05 ng of DNA), we detect only 2 peaks corresponding to 12S and SWIM amplicons. This means that our PCR sexing assay is highly specific (Fig 1C). In term of sensitivity, the SWIM/12S assay is able to detect low concentrations of DNA since it amplifies SWIM and 12S amplicons from crude extracts containing only 1 ng of total DNA (Fig 1A).

To sex large number of chicken eggs, the sexing method needs to be simple and rapid without extensive and expensive purification and quantification steps. To this aim, we established a very simple extraction protocol with one incubation step with beads that chelate potential PCR inhibitors followed by a centrifugation step. We adapted this extraction protocol to analyse crude extract from different tissues of an egg including outer membrane, inner membrane, chorion, blood and embryo without DNA quantification (Table 2).

**Table 2:** PCR sexing accuracy at different development stages for various tissues.

Tissue →	Shell				Outer membrane				Inner membrane		Albumen		Chorion		Blood		Embryo tissue	
	Air Chamber Side		Opposite side		Air Chamber Side		Opposite side		Air Chamber Side									
Chelex Digestion →	Yes	No	Yes	No	Yes	No	Yes	No	Yes	No	Yes	No	Yes	No	Yes	No	Yes	No
Incubation Day ↓																		
0	-	-	-	-	-	-	-	-	-	-	-	-	-	-	-	-	-	-
2	-	-	-	-	-	-	-	-	-	-	-	-	++	-	-	-	-	-
4	-	-	-	-	-	-	-	-	+	-	-	-	++	-	+	-	+	-
6	-	-	-	-	+	-	+	-	++	-	-	-	++	-	++	-	++	-
9	-	-	+	-	+	-	++	++	++	++	+	-	++	++	++	+	++	+

Table summarizing the accuracy of our SWIM/12S PCR sexing method on different chicken egg tissues. **-**: gene not detected;

**+**: gene detected with low yield or maternal contamination;

**++**: gene detected with high yield and high specificity. The new assay provides reliable sexing result even in highly heterogeneous samples with unknown DNA concentrations. For more details about the amount of tissues used and extracted DNA see S2 Table.

To verify if the newly designed PCR sexing method could cover genetic diversity among different chicken breeds, samples from 4 different races (Shaver black, Dekalb white, Isa brown and Bovan brown) were examined (Fig 1B). The versatility of the assay among chicken breeds was confirmed since the PCR clearly generates a single 131 bp band for male embryos and two bands (131 bp and 212 bp) for the females.

In addition, among avian, the multiplexed SWIM/12S PCR sexing method is specific to chicken sex identification since SWIM primers are only able to anneal on *Gallus gallus* genome. In order to rule out the possibility for the SWIM and 12S primers to amplify other birds or human DNA, we performed PCR amplification with the SWIM and 12S primers in the presence of human, duck, pigeon and guinea fowl DNA (S5 and S6 Figs). These experiments show that there is no amplification of other birds or human DNA with our PCR assay. This aspect is clearly an advantage if sexing has to be performed in an environment with high risk of contamination such as hatchery.

#### Evaluation of the newly designed assay in comparison with the existing PCR methods

**Fig 2:**
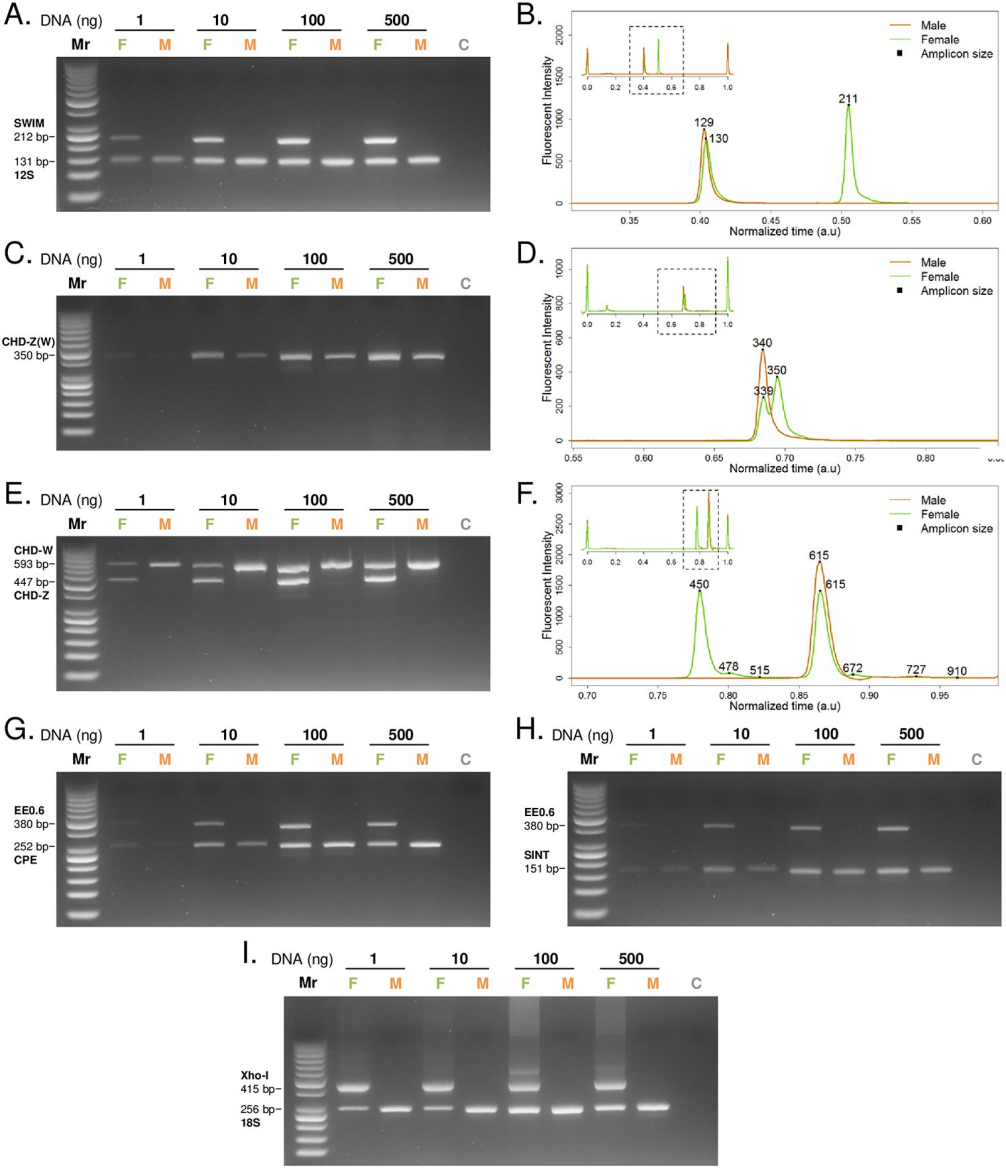
Performance of the newly designed assays in comparison with previously described chicken PCR sexing methods. (A, C, E, G, H, I) Agarose gel electrophoresis of PCR amplification from extracts of ISA brown chicken embryo using different primer sets: A. SWIM/12S, C. CHD P2/P8, E. CHD 2250F/2718R, G. EE0.6/CPE, H. EE0.6/SINT, I. XhoI/18S. 1 to 500 ng of either female or male DNA was used in each reaction (M: Male. F: Female. C: Negative control. Mr: 50 bp ladder markers). (B, D, F) Labchip analysis of the corresponding PCR (one female and one male) with different set of primers: B. SWIM/12S, D. CHD 2250F/2718R, F. CHD P2/P8 (500ng DNA input).

We next compared the specificity, sensibility and robustness of the newly designed assay to those of previously described methods. We chose some of the most commonly used PCR-based assays for avian sexing (CHD-1 (P2&P8), CHD-1 (2250F/2718R), EE0.6 & CPE, EE0.6 & SINT, XhoI & 18S [4, 6–8, 10]) to address their performances as reference assays. PCR reactions were performed with different DNA concentrations (1–500 ng) both for newly designed and reference primers. PCR amplification was analysed by both agarose gels and microfluidic capillary electrophoresis [20] (Fig 2).

Sex-linked homolog genes—CHD-1 (single primer pair): The chromo-domain helicase DNA binding 1 (CHD-1) was the first gene proposed to differentiate a wide range of non-ratite bird species [6, 7, 21]. CHD-1 is located on both Z (CHD-Z) and W (CHD-W) chromosomes with the presence of some polymorphisms, allowing their specific detection by PCR amplification. Several studies explored these differences to design multiple specific primers [4, 7, 22]. To compare our method to CHD-1 assays, we reproduced CHD1-linked experiments using P2/P8 [7] and 2250F/2718R primers [6] (Fig 2C and 2E). According to the study concerning the P2/P8 CHD-1 DNA amplification, we are able to depict 2 different population of amplicons only when microfluidic capillary electrophoresis was used as detection method (Fig 2D). The regular method using agarose gel electrophoresis does not allow depicting the 10 base pair difference between the 2 amplicons (Fig 2C). Griffiths et al. suggest using an 8% denaturing acrylamide gel to resolve this problem, which is less convenient than agarose gels to sex a large number of chicken eggs. Moreover, the small difference of only 10bp between the 2 amplicons can easily lead to misinterpretation.

Another CHD-1 gene sexing method using primer pair called 2250F/2718R [6] was examined. This mono-pair primer designed assay generates one male and one female specific fragment with a difference of 147 base pairs (Fig 2E). However, the CHD-1 2250F/2718R assay generates a competition between the two amplicons when DNA input was in between 1 and 10 ng range. Indeed, the intensity of the control band (male band) diminishes once the female specific band is amplified. When more DNA input was used, for 100–500 ng range, unspecific amplification occurs, which can lead to misinterpretation on large scale sexing (Fig 2E and 2F). The newly designed SWIM assay shows its advantages over CHD-1 assay as generating neither competition between amplified amplicons nor unspecific amplification in all tested conditions.

W-linked gene with ribosomal gene as control: XhoI + 18S: Several PCR sexing methods based on the detection of one W-linked gene and one ribosomal gene as a control were previously developed. Among them we chose to evaluate the assay that amplifies XhoI and 18S genes [4] (Fig 2I) in which XhoI is a repetitive DNA sequence located on W chromosome of *Gallus gallus* domestic fowl [23]. Compared to our SWIM/12S assay, the XhoI/18S assay is not advantageous as it generated multiple amplicons together with a smear in the large molecular mass rang. This noisy amplification profile is probably due to the recognition of the XhoI primers on multiple sites of the repetitive region on the chromosome W. Moreover, like the CHD-1 assay, XhoI/18S method also generates a competitive amplification between the two amplicons (Fig 2I).

W-linked gene with a Z-linked sequence as control: EE0.6 + SINT & EE0.6 + CPE: Other types of assays, based on the specific detection of one gene on W chromosome and the other on Z chromosome, have been developed to sex chicken. Among them, we decided to test the accuracy of the EE0.6 + SINT and EE0.6 + CPE PCR assays (Fig 2G and 2H). EE0.6 is conserved between bird species [11, 24] allowing female determination of different birds combined with Z-linked Spindling gene sequences (CPE, SINT) as control (male indication) [10, 11, 24, 25]. Three primer sets have been used to reproduce these assays: W-linked EE0.6, Z-linked SINT and Z-linked CPE. The female specific EE0.6 primers were designed to amplify a non-repetitive DNA sequence within the EcoRI region of the W chromosome. Z-linked SINT and CPE primers amplify Spindling genes on chromosome Z of chicken specifically [26]. Both two methods have a good specificity since no extra-amplicon was detected. However, these two methods are less sensitive than the newly designed SWIM/12S assay, since PCR amplification is very low at 1 ng DNA input.

Based on the obtained results and the literature, we summarized the performance of all tested assays in Table 3. In summary, to specifically assess chicken sex, our new designed method SWIM/12S has the best score combining specificity, sensitivity and high-throughput applicability beyond all the 6 tests.

**Table 3:** The main general attributes of PCR methods used in the sex identification of birds.

Methods								
Primers↓	W-Linked genetarget	Control genetarget	Specificity	Sensitivity	Reprodu-cibility	High-throughputapplicability	Intensiveness of labor	Ref.
**SWIM/12S**	SWIM	12S	+++	+++	+++	+++	Low	This study
**P2/P8**	CHD1W	CHD1Z	-	-	-	-	Moderate	[7]
**2250/2718**	CHD1W	CHD1Z	++	+++	++	++	Moderate	[6]
**XhoI/18S**	Xho-I	18S	+	+++	++	++	Moderate	[4]
**EE0.6/CPE**	EE0.6	CPE	+++	+	++	++	Moderate	[10]
**EE0.6/SINF**	EE0.6	SINF	+++	-	++	++	++	[10]

-: fair; +: good, + +: very good; + + +: excellent. The table was modified from F. Morinha [20].

#### Large scale assessment of SWIM/12S PCR assay

**Fig 3:**
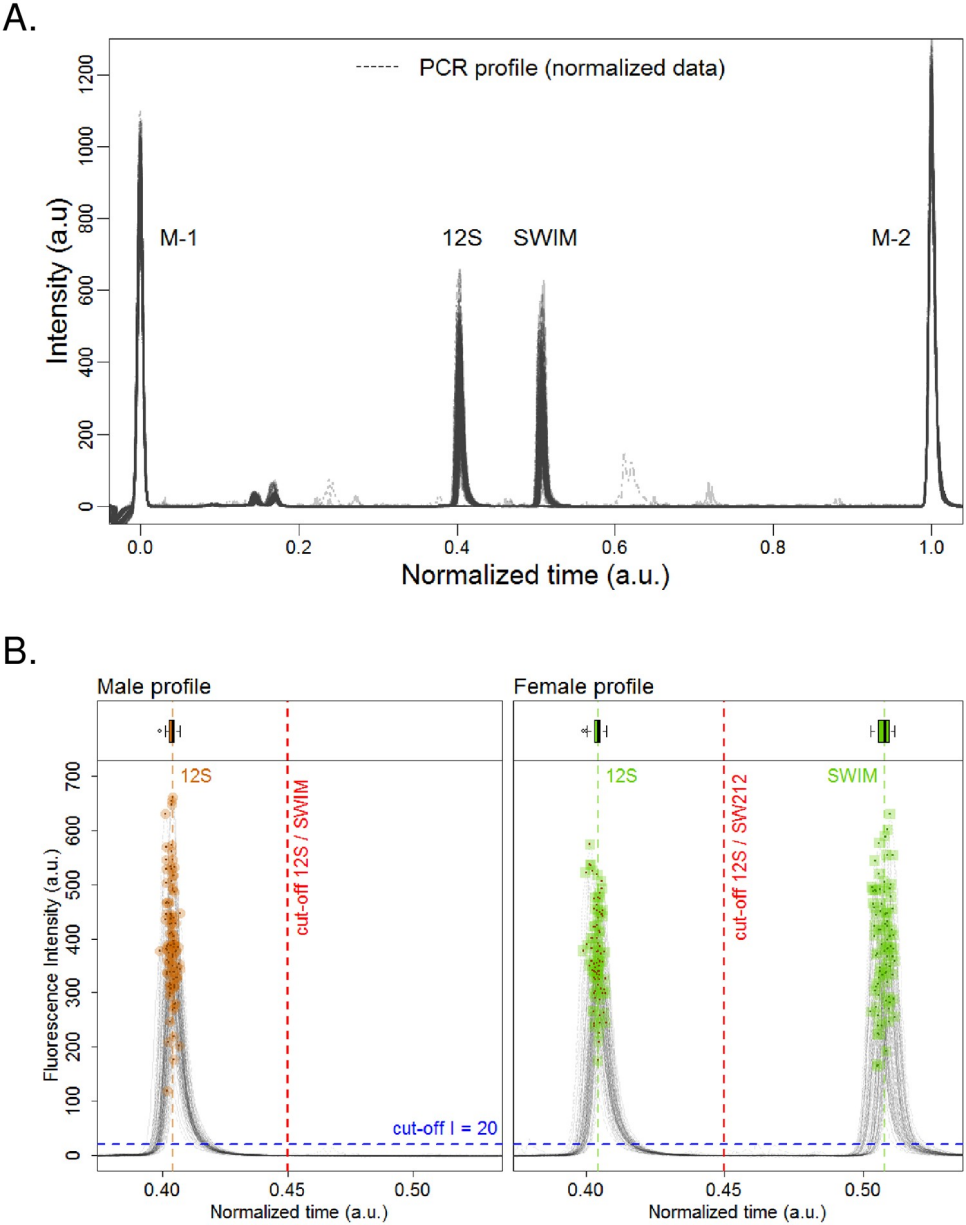
Robustness assessment of the SWIM/12S PCR assay. Analysis by capillary electrophoresis. (**A**) Superposition of the 176 electropherograms corresponding to the analysis of the 176 chicken embryo crude extracts. 2 markers were used to normalize the migration time of the PCR products (M-1: Marker-1 at the beginning of the measurement, M-2: Marker-2 at 1500bp). (**B**) All values of males’ and females’ peaks were highlighted with orange and green point, respectively. All tested female samples generated two peaks (SWIM: 212 bp and 12S: 131 bp), while male samples gave only one peak at 131 bp (12S). The upper panel contains three boxplots representing the distribution of the data.

After demonstrating that the SWIM/12S assay designed in our study is more advantageous over all other tested assays, we decided to validate the robustness of our new assay in a large scale analysis. To this aim, we conducted a study on a large number of samples using SWIM/12S primers and CHD 2250F/2718R primers as reference for sex determination [6]. 176 Isa Brown chicken embryos’ samples were tested and DNA amplification was analysed by capillary electrophoresis (CE). We took advantages of the CE system in terms of speed, high-throughput applicability, automated workflow, resolution, and sensitivity compared to conventional slab gel electrophoresis (Fig 3A). For robustness assessment, the crude embryo extracts was directly used for PCR amplification without DNA quantification and purification to preserve a high heterogeneity among samples in term of DNA concentration and contaminants.

Among the 176 embryos analysed, we identified 80 females with 2 peaks on the electropherogram and 94 males with only 1 peak. Due to the heterogeneous nature of the samples in term of DNA concentration, the fluorescence intensity varies between samples but the position of the 2 peaks (SWIM and 12S) is highly stable (Fig 3B). Among the 176 samples, 4 of them led to the amplification of minor unspecific DNA population but it did not affect their sex identification (Fig 3A). Embryos sexing obtained with our new SWIM/12S assay are 100% concordant with the reference assay using 2250F/2718R primers [6]. Two samples were considered as invalid since the signal to noise was not good enough. To follow up this invalidation, we did the dosage of these 2 samples and found that DNA concentration was below 0.05 ng. We decided to redo the PCR reaction for these samples using 2 ng DNA. At this point, we were able to assign a valid sex for these 2 samples.

Based on these results, we concluded that the SWIM/12S PCR sexing assay represent a reliable, robust and effective method for chicken sexing.

### A new robust Q-PCR sexing method for high scale use

**Fig 4:**
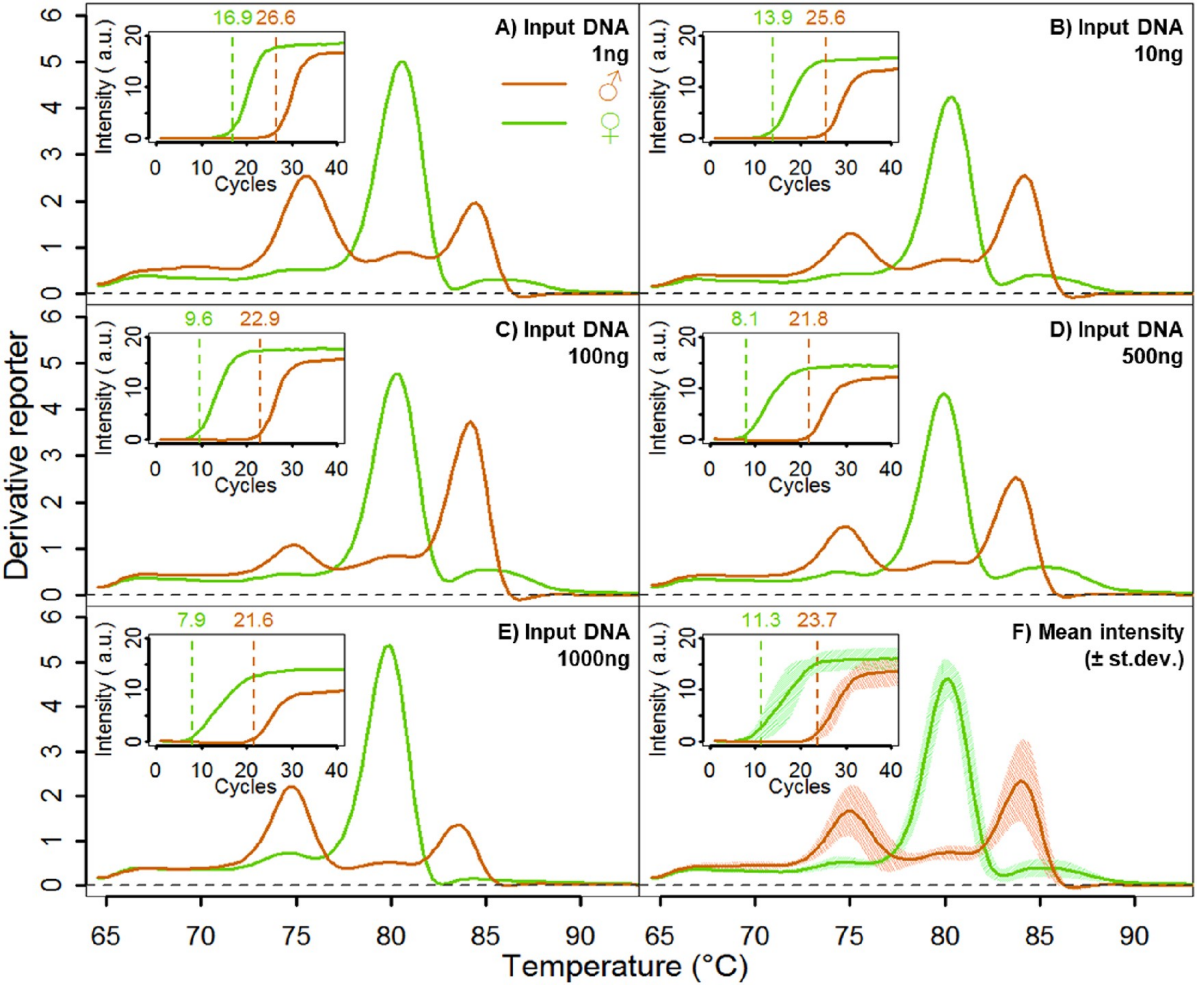
Sensitivity of our DMRT/Xho-I Q-PCR assay. (A-E) CPA and MCA analysis of known sex ISA brown chicken (Green: Females, Orange: Males). Chicken embryo extracts containing 1 to 1000 ng DNA were used here (**A**: 1 ng, **B**: 10 ng, **C**: 100 ng, **D**: 500 ng and **E**: 1000 ng). For CPA analysis, fluorescence intensity was measured at each cycle and is represented in the upper left graph for each DNA concentration. Melting curves were obtained between 65°C and 95°C. (**F**) Mean melting curve profiles for females (Green) and males (Orange) chicken at 1 to 1000 ng DNA content. Mean Tm values +/- standard deviation are shown on the graph.

The Real Time quantitative PCR (Q-PCR) allows high-throughput DNA identification since it is fast and does not require electrophoresis based DNA migration. This method therefore has been used widely for chicken sexing [3, 5, 22, 27]. However, it has not yet been validated in term of sensitivity, specificity and robustness at large scale in a high throughput format. To this aim, we designed and tested 60 pairs of primers for their ability to sex chicken embryos by Q-PCR under such constrains. Among them, we identified 2 pairs of primers amplifying the DMRT gene on the Z-chromosome and the Xho-I repeated region on the W-chromosome as potential elements for our Q-PCR sexing assay when combined the analysis of the crossing point (CPA) and of the melting curve (MCA). We first investigated the sensitivity of the reaction by looking at the effect of different embryo template DNA concentration on the Q-PCR reaction to determine if we could eliminate the DNA quantification step (Fig 4). 5 concentrations of males and females’ chicken DNA from 1 ng to 1 μg were tested (Fig 4A–4E).

Our results show that the DMRT/Xho-I Q-PCR assay allows a clear differentiation between males and females chicken from very low crude extracts DNA concentration to high concentrations. Into detail, we depicted a Cp difference of more than 10 cycles between female and male samples containing between 1 ng and 1 μg DNA (Fig 4). This Cp analysis constitutes the first step of our discrimination assay. Taking into account the variability of Cp at different concentration we observed that with a cut-off Cp value of 18 we can securely discriminate between males and females at these concentrations.

**Fig 5:**
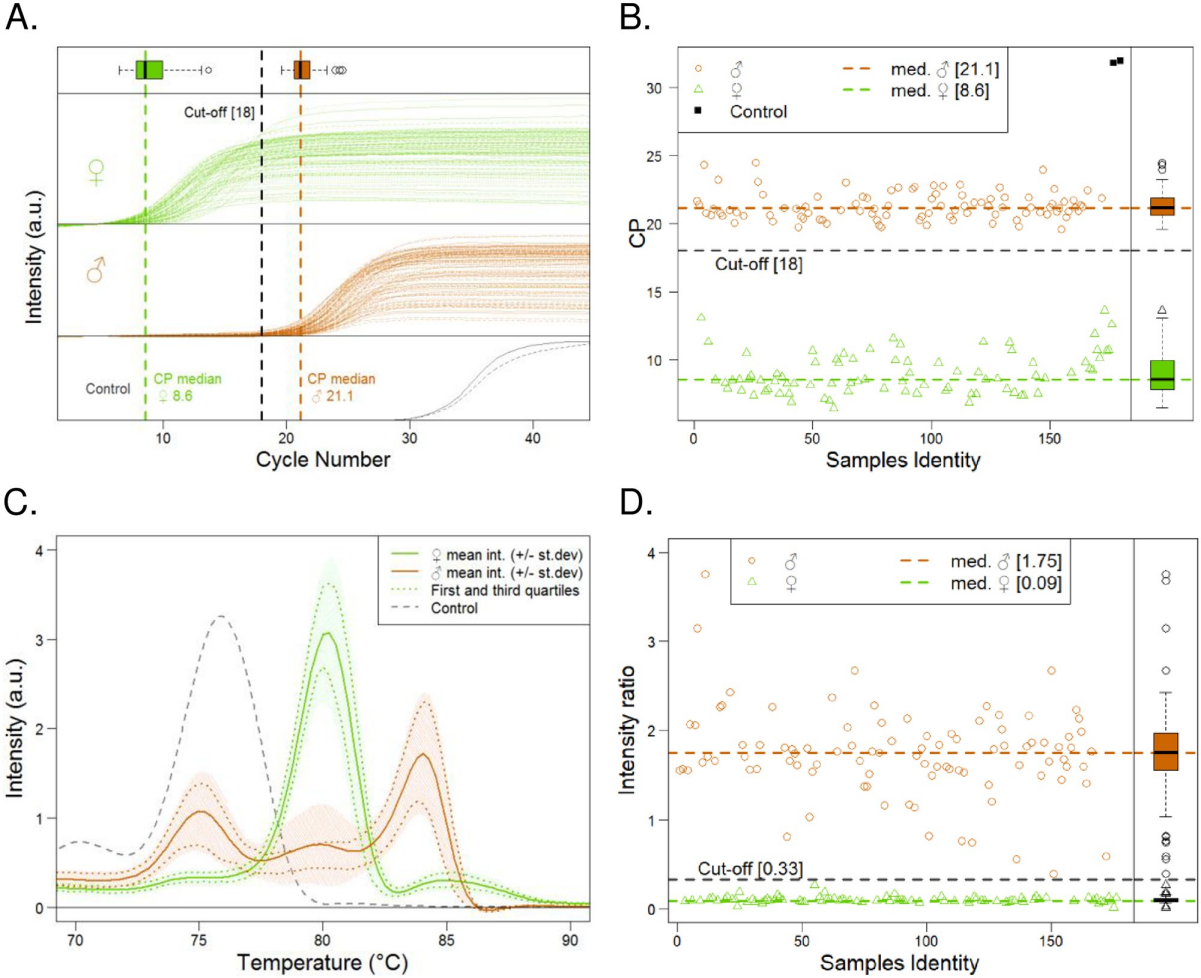
Robustness analysis of the DMRT/Xho-I Q-PCR assay. 176 chicken samples sexed by conventional PCR method with primers 2250F/2718R [6] were used. Among them, 97 males (♂- Orange) and 84 females (♀- Green) were tested. 2 negative controls containing no DNA were included (**A, B**); Crossing point analysis (CPA) (**C, D**); Melting curve analysis (MCA). (**A**) Fluorescence signal of Q-PCR amplification for males (Orange), females (Green) and negative controls (Grey). (**B**) Cp values analysis of the 176 samples. Boxplots represent the statistical distribution of females and males’ CPA. (**C**) Mean Tm curves for females and males samples. Statistical distribution was represented by mean value, first and third quartiles +/- Standard deviation. (**D**) Ratio of Tm peak value 84°C/79°C for the 176 samples.

As a second step, to reinforce the Cp discrimination, we compared the melting temperature (tm) profiles at the different DNA concentrations. As shown in Fig 4A–4E, female chicken exhibit one single peak around 80°C, whereas males show two peaks around 75°C and 84°C. We decided to use the ratio of the intensities between 80°C and 84°C to discriminate males and females chicken (Fig 5D).

#### Q-PCR test performance assessment

In order to evaluate our Q-PCR method, we tested its reproducibility and robustness on 176 sexed embryos without DNA quantification and purification. Average Cp was 8.6 for females, 21.1 for males and 32 for negative control (Fig 5A). By defining a Cp value cut-off at 18, we were able to clearly discriminate between males and females with 100% concordance. The averages Tm ratios of 84°C/80°C intensities were 0.033 ±0.1 for females, 1.76 ±0.5 for males (Fig 5B). A 84°C/80°C ratio cut-off at 0.33 lead to 100% of discrimination between males and females.

To address the reproducibility, we prepared 2 different extractions for each 176 embryos for sex determination by the designed Q-PCR. First, Cp and Tm analysis were 100% concordant and we got 100% concordance between the 2 sets of experiment for each of the 176 embryos. In addition, these Q-PCR results were 100% concordance with the sex assessment and the PCR using CHD assay. Thus, we developed a new simplified Q-PCR based sexing method, which does not require DNA purification and quantification in the first steps. This subsequently leads to a gain of time and money when large number of analysis is required both for the industry and the scientific community.

#### Comparison with the existing Q-PCR methods

Different Q-PCR based methods have been developed to sex chicken. Based on literature studies, we compared our method to the most used ones (Table 4). Among the criteria, we considered the accuracy, the sensitivity and the time and money costs of the methods.

**Table 4:** Comparative analyses of the main advantages and limitations of existing Q-PCR sexing methods.

Method name (1^st^ Author)	Our method	Rosenthal[22]	Chen[3]	Morinha[27]	Clinton[5]
Targeted genes (*)	DMRT / Xho-I	CHD-1	CHD-1	CHD-1 (P2/P8 region)	Xho-I / CR1
**Detection method**	Sybr green	Taqman	Sybr green	Evagreen	FRET Cassette
**Parameters** **Analysed**	CPA / MCA (Cycles / Melt. Curve)	CPA (CT)	MCA	MCA	Quenching release
**Accuracy**	+++	++	+	++	+++
**Number of reactions necessary**	1	1	2	1	1
**Necessity of quantification of input DNA**	No	No	Yes	Yes	No
**Sensitivity / Reproducibility**	+++	++	+	++	+++
**Minimum input DNA**	1ng	1ng	5 ng	20ng	1ng
**Number of samples tested**	176	408	18	111	500
**Robustness assessed in the paper**	Yes	No	No	Partially	Yes
**Column DNA extraction (DNeasy Kit Qiagen)**	No	No	Yes	Yes	No
**Experiment time-cost**	~2 hours	~2 hours	~3 hours	~3 hours	~1 hour
**Estimated consumable cost per reaction**	0.3$	~2$	~3$	~2$	~2$
**Avian Specificity**	*G*. *gallus* specific	*G*. *gallus* specific	*G*. *gallus*, *P*.*Chinensis* tested	14 avian species tested	*G*. *gallus* specific

For more details about the cost estimation, please see S3 Table.

*Primers are different for the targeted gene CHD-1 between Chen, Rosenthal and Morinha studies.

Q-PCR sexing based on TaqMan probes with CT (threshold cycle) analysis: This approach developed in 2010 is based on the threshold cycle analysis of Q-PCR reactions [22]. It has a good sensitivity and specificity but is very expensive due to the probes needed for the detection of males and females specific amplicons. It is a disadvantage for large scale analysis.

Sybr Green Q-PCR method based on CHD1 amplification: In 2012, a Sybr Green Q-PCR method based on CHD1 amplification was proposed to sex chicken [3]. This approach was based on the melting curve comparison (MCA) between males and females chicken tissues. It is effective for sex identification but it needs DNA purification and two distinct Q-PCR reactions with specific primers for female and male. This is time and cost consuming, which again, is a strong weakness in the context of simplified and large scale analyses.

Q-PCR sexing combined with High Resolution Melting analysis (HRM): Q-PCR combined with High Resolution Melting analysis (HRM) has also been adapted for chicken sex identification [27]. HRM is an optimized MCA analysis that allowed the discrimination of amplified product with less than 10 bp difference (like P2/P8 amplicons). Despite, its high accuracy, the main negative aspect is the requirement of beforehand DNA enrichment and dosage. This increase the number of steps making it time and cost consuming.

FRET probes assay adapted for chicken sexing from Clinton et al: In 2016, a FRET probe based assay has been used for chicken sexing [5]. They used the invader approach by targeting Xho-1 and CR1 genes with specific FRET probes. This method has the advantage to be effective at isothermal temperature (63°C) without the need of thermocycler. This technique seems highly specific, sensitive and fast but the main disadvantages remains its high cost compared to a regular Q-PCR.

Compared to all these existing methods that already show relevant performances, our newly designed Q-PCR DMRT/Xho-I assay combines all the advantages suitable to simplified, reduced cost and high throughputs assays by keeping a very high sensitivity and specificity. In particular, our assay requires a reduced number of steps (neither DNA purification nor DNA quantification) leading to efficiency improvement and time saving. Consequently, our assay is time and cost saving reaching the constraints for further large scale studies.

## Conclusions

The scientific community and the poultry industry lack a reliable method to determine chicken embryo sex for which the performances have been evaluated and validated in a large scale manner. Indeed, benchmarking new technologies aiming to sex chicken eggs in a non-invasive way, will need a highly-reliable invasive method as reference (gold standard) to determine the sex of the embryo a posteriori that works at early stage of embryo development. Here, we developed two new simple alternative methods to sex chicken embryos and demonstrated their high performance for large-scale purpose. The 2 methods exhibit 100% of concordance and specificity for the sex determination of the 176 embryos tested in our studies. One of the methods is based on PCR technologies once the other one is based on RT qPCR. Depending on their resources and equipment, the users, in a scientific lab or in the poultry industry can choose between the 2 approaches. For users equipped with real-time PCR readers the RT qPCR will be more convenient since this approach requires fewer steps than PCR and the results analysis can be easily automatized. PCR will be more convenient for users that do not want to invest too much money in the equipment. Compared to the other technologies already developed to sex chicken, our testes combine high compliancy with large scale analysis, cost effectiveness and high sensitivity. Moreover, we showed that our testes are efficient on many different tissues of the embryo and can be used for different chicken breeds in a short-time scale since the 2 methods do not require any DNA purification step. Consequently, any laboratory from any speciality can easily implement our simplified and robust sexing assays using the Standard Operating Procedures (SOP) provided on protocols.io (dx.doi.org/10.17504/protocols.io.uf6etre). The positive results from experiments with membrane samples (external and internal) from which the embryos continue to develop encourage a future development for a semi invasive *in ovo* sexing method.

## Supporting information

S1 Fig Primer couples tested for our PCR test. Control and Female specific primers: SWIM, 12S, CHD-P8F/P2R, DMRT2 NCB343, HTW-10, DMRT2’-NCBI82, FOX8-NCBI91, DMRT2’-NCBI57.
(TIF)

S2 Fig Primer couples tested for our PCR test. Control and Female specific primers: HTW4’, HTW3-R1, FET1-NCBI786, HTW3-R2, DMRT2’-NCBI343, HTW-NCBI879, HTW-NCBI689, HTW-NCBI731.
(TIF)

S3 Fig Primer couples tested for our PCR test. Control and Female specific primers: HTW2-F2, HTW2, FET1-NCBI743, FOX8-NCBI166, FOX8-NCBI109, FOX8, 18S-5, XhoI-7.
(TIF)

S4 Fig Primer couples tested for our PCR test. Control and Female specific primers: EE06, CHD5, Sinf, Cpe, RAS326, RAS400, RAS454, WC430.
(TIF)

S5 Fig Specificity of the SWIM / 12S PCR test for chicken versus Human DNA. The SWIM / 12S PCR test is specific for chicken versus Human DNA. DNA extracted from human SH-S5Y cells is not amplified by the SWIM / 12S PCR test. Hum: Human DNA extract; F: Chicken female DNA extract; M: Chicken male DNA extract; NT: No DNA template. FAM192A: Primers specifics for human DNA amplification.
(TIF)

S6 Fig Specificity of the SWIM / 12S PCR test for chicken versus duck, guinea fowl and pigeon DNA. The SWIM / 12S PCR test is specific for chicken versus Duck, Guinea fowl and Pigeon DNA. DNA was extracted from adult anatomically confirmed sex birds. Duck, Guinea fowl and Pigeon brain DNA is not amplified by the SWIM / 12S PCR test whilst those from adult chicken are amplified. F: Female DNA extract; M: Male DNA extract; NT: No DNA template. DNA was extracted from brain tissues for all the different birds as described in materials and methods.
(TIF)

S7 Fig Agarose gel electrophoresis profile of DMRT / Xho I QPCR amplification. The DMRT / Xho I QPCR amplification profiles of males and females DNA on Agarose gel electrophoresis is clearly different. It explains the Tm profiles difference for males and female. 3 independent experiments were performed and show the same result.
(TIF)

S1 Table Detail of the primers tested for the development of the PCR assay. (TIF)

S2 Table Amount of DNA extracted from the various tissues tested. Table summarizing the amount of tissues used for each DNA extraction (in mg) and amount of DNA extracted from each tissue (in ng/mg of tissue).
(TIF)

S3 Table Detail of the cost estimation of the different Real-Time PCR sexing methods. (TIF)
