# Supplementary figures and images for: Simple, sensitive and robust chicken specific sexing assays, compliant with large scale analysis
Source: PLoS One. 2019 Mar 1;14(3):e0213033. doi: 10.1371/journal.pone.0213033 (PMC6396912; doi:10.1371/journal.pone.0213033)

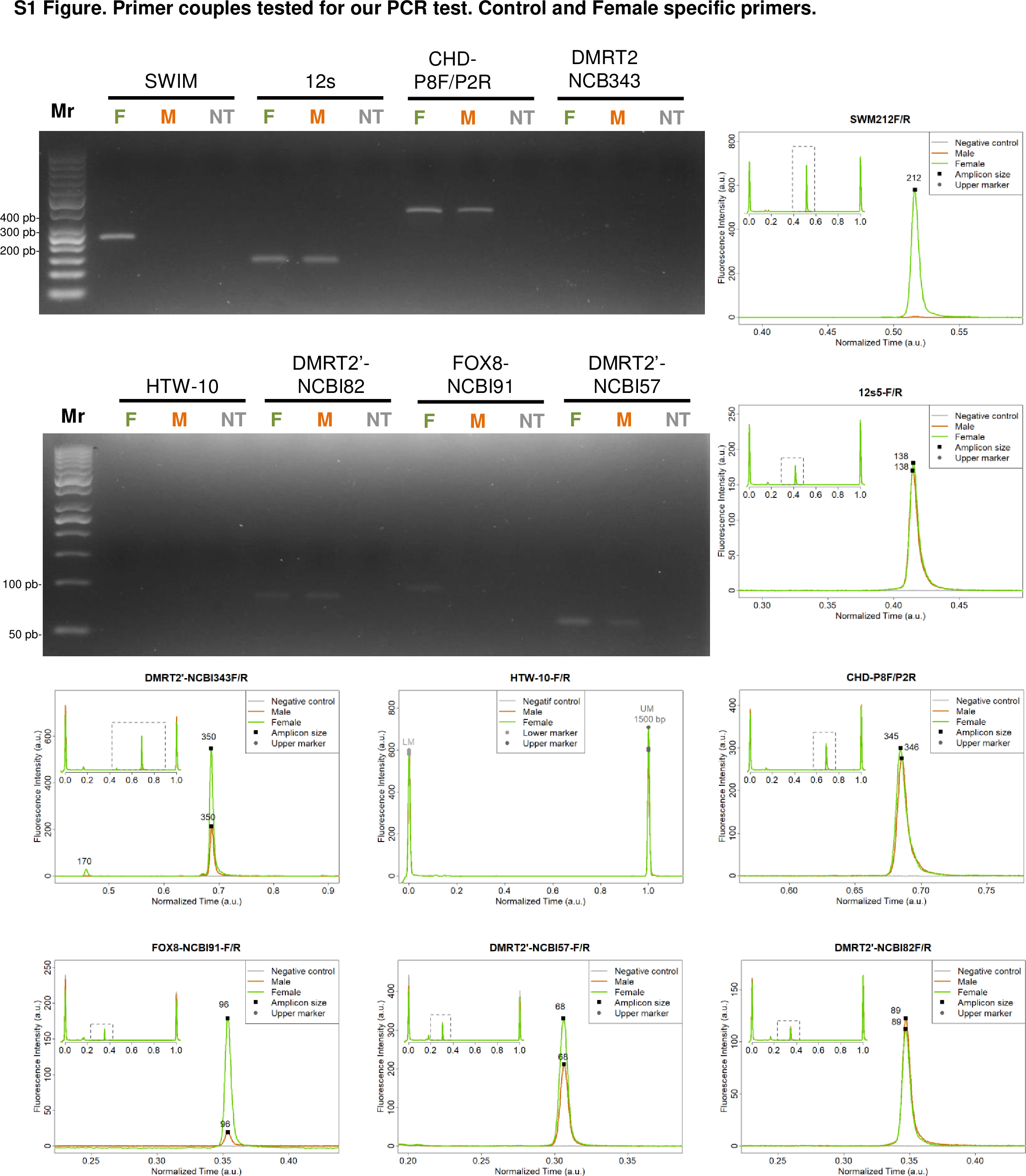

Supplement: S1 Fig — Control and Female specific primers: SWIM, 12S, CHD-P8F/P2R, DMRT2 NCB343, HTW-10, DMRT2’-NCBI82, FOX8-NCBI91, DMRT2’-NCBI57. (TIF) [file pone.0213033.s001.tif]

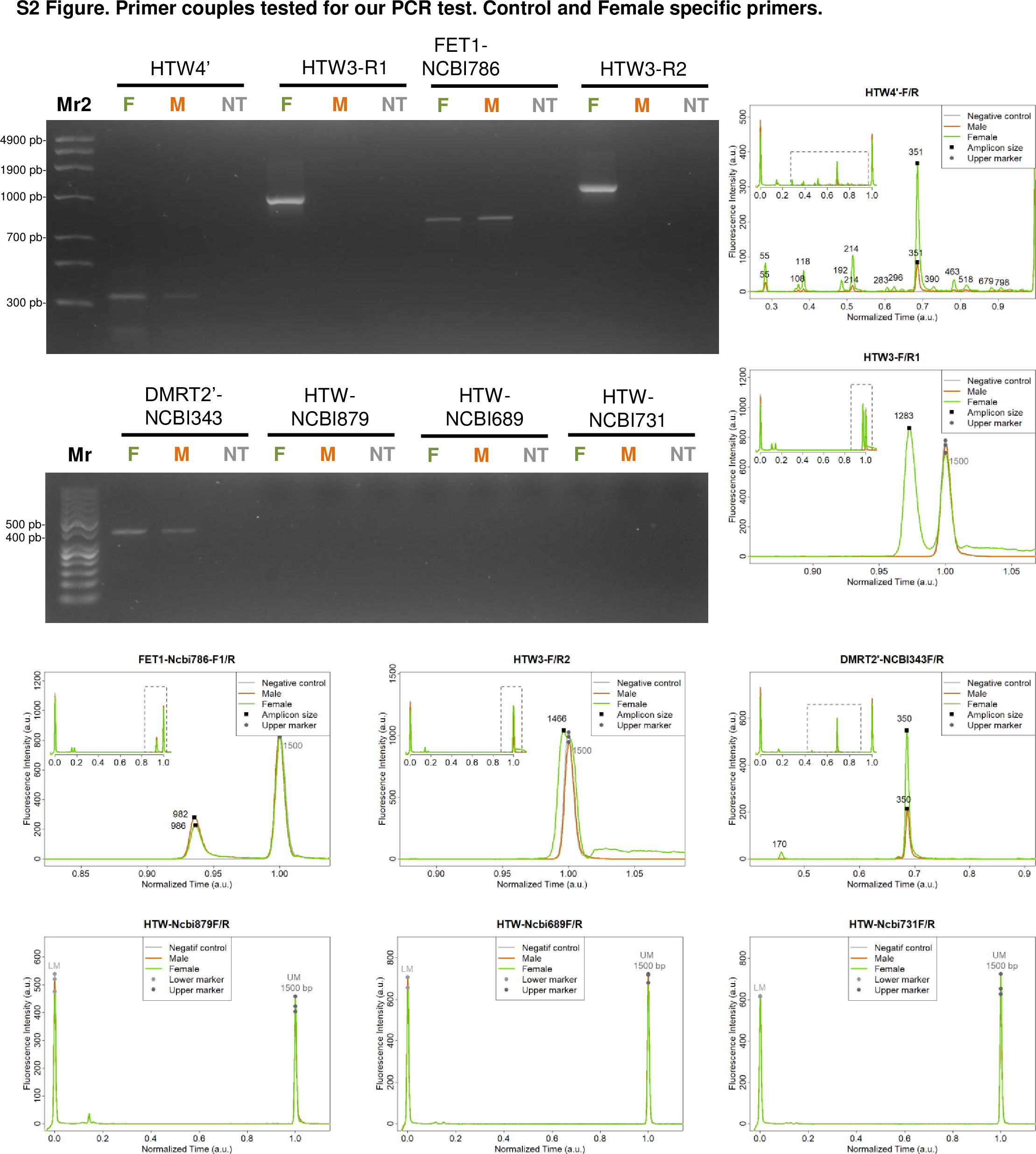

Supplement: S2 Fig — Control and Female specific primers: HTW4’, HTW3-R1, FET1-NCBI786, HTW3-R2, DMRT2’-NCBI343, HTW-NCBI879, HTW-NCBI689, HTW-NCBI731. (TIF) [file pone.0213033.s002.tif]

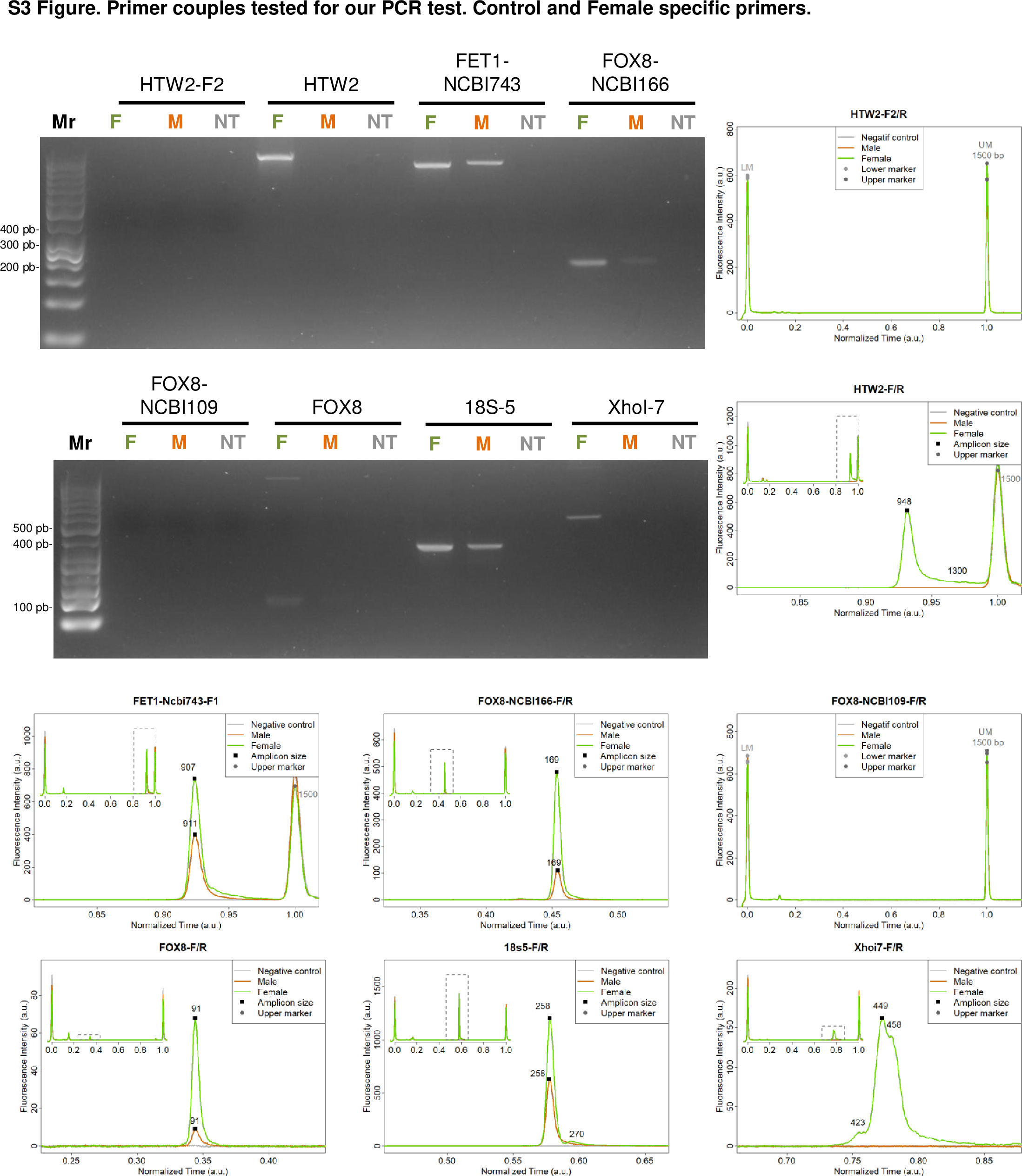

Supplement: S3 Fig — Control and Female specific primers: HTW2-F2, HTW2, FET1-NCBI743, FOX8-NCBI166, FOX8-NCBI109, FOX8, 18S-5, XhoI-7. (TIF) [file pone.0213033.s003.tif]

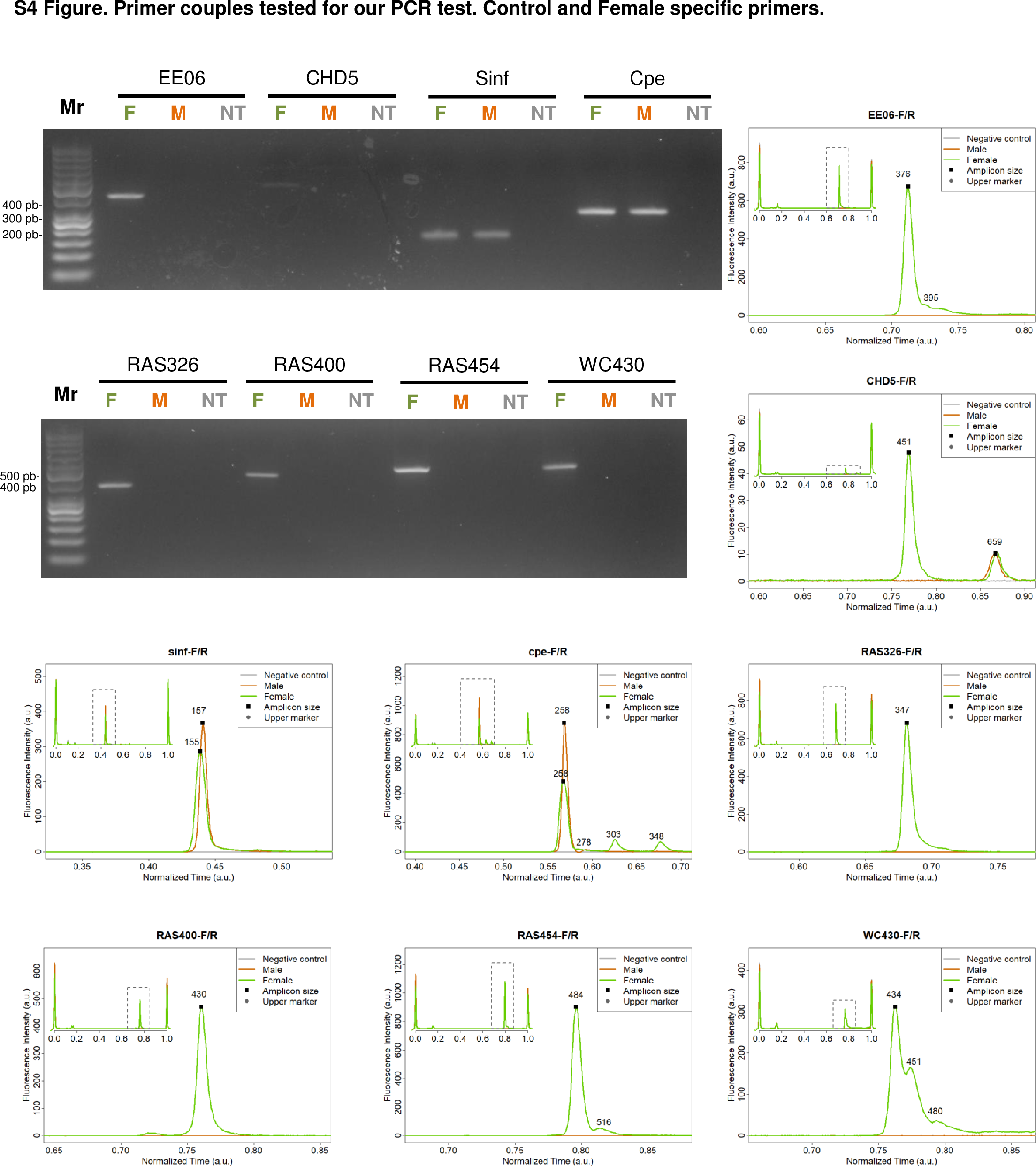

Supplement: S4 Fig — Control and Female specific primers: EE06, CHD5, Sinf, Cpe, RAS326, RAS400, RAS454, WC430. (TIF) [file pone.0213033.s004.tif]

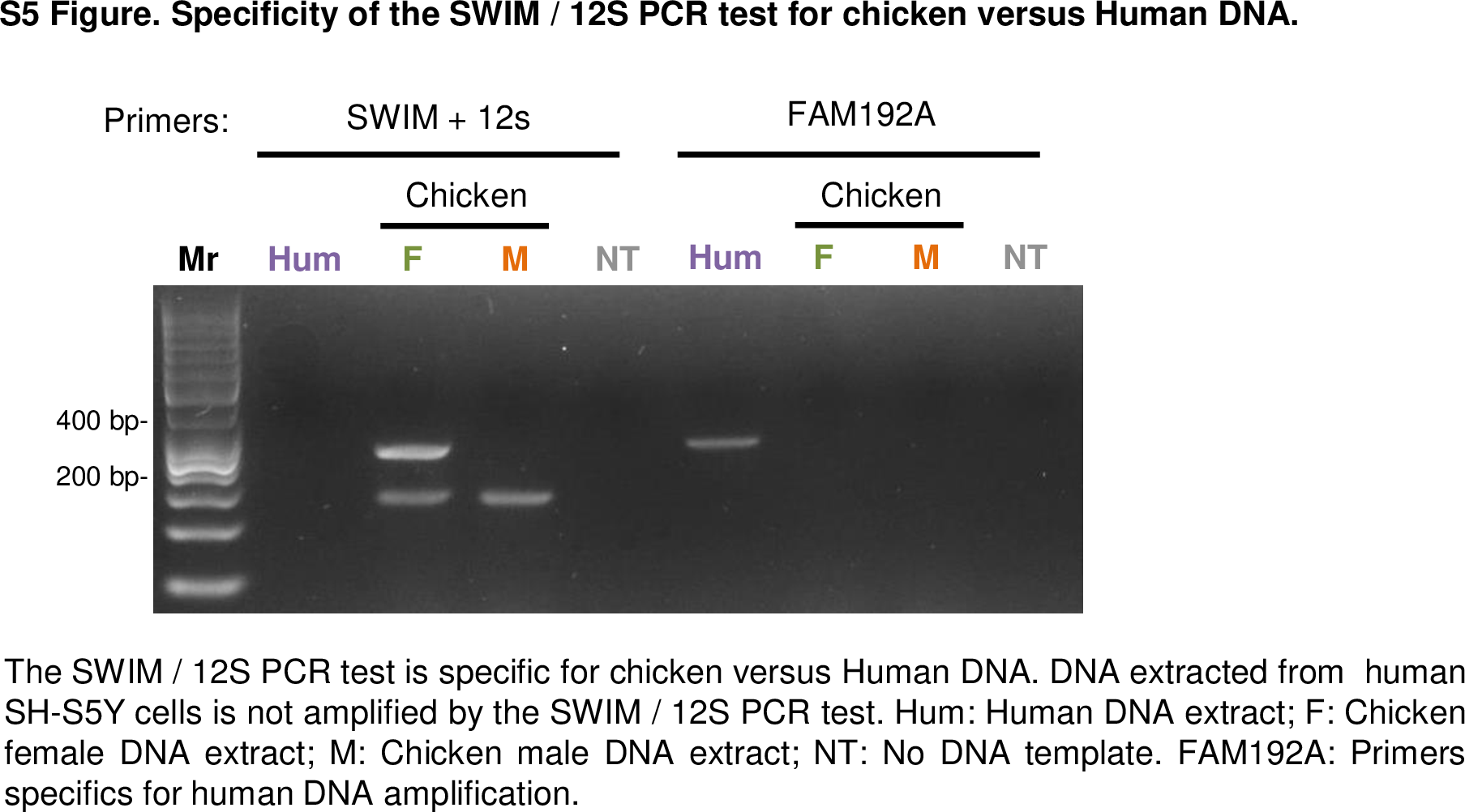

Supplement: S5 Fig — The SWIM / 12S PCR test is specific for chicken versus Human DNA. DNA extracted from human SH-S5Y cells is not amplified by the SWIM / 12S PCR test. Hum: Human DNA extract; F: Chicken female DNA extract; M: Chicken male DNA extract; NT: No DNA template. FAM192A: Primers specifics for human DNA amplification. (TIF) [file pone.0213033.s005.tif]

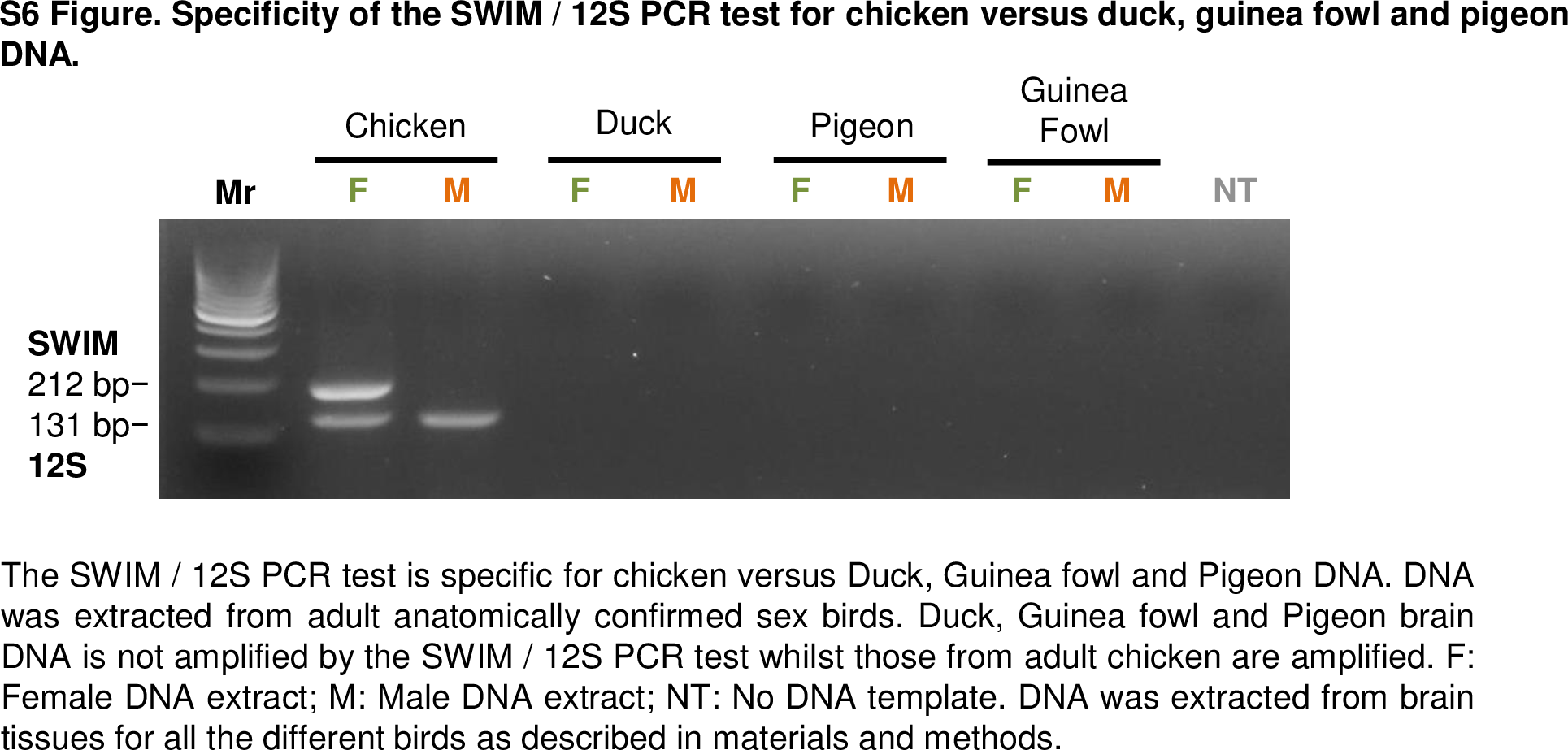

Supplement: S6 Fig — The SWIM / 12S PCR test is specific for chicken versus Duck, Guinea fowl and Pigeon DNA. DNA was extracted from adult anatomically confirmed sex birds. Duck, Guinea fowl and Pigeon brain DNA is not amplified by the SWIM / 12S PCR test whilst those from adult chicken are amplified. F: Female DNA extract; M: Male DNA extract; NT: No DNA template. DNA was extracted from brain tissues for all the different birds as described in materials and methods. (TIF) [file pone.0213033.s006.tif]

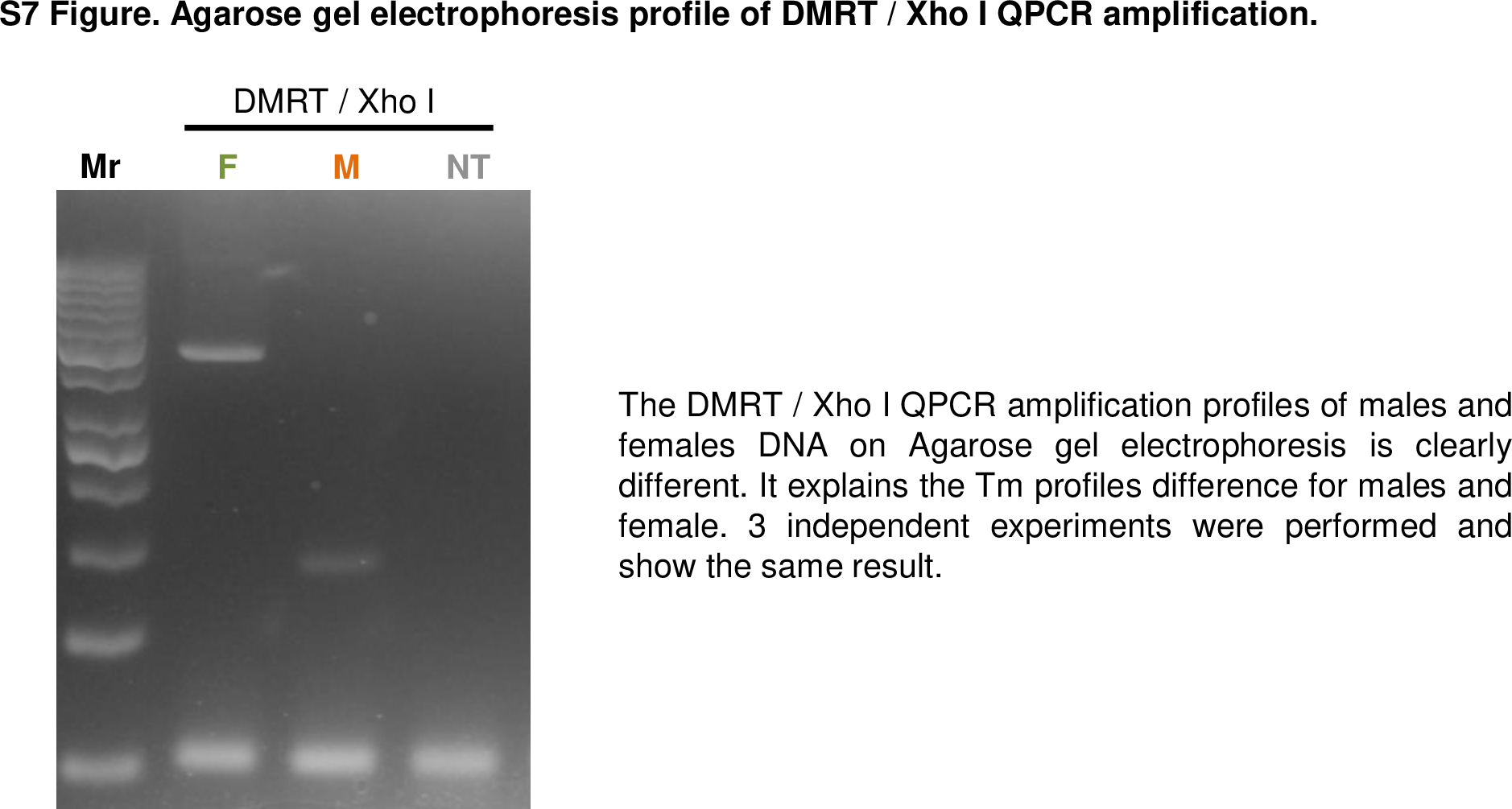

Supplement: S7 Fig — The DMRT / Xho I QPCR amplification profiles of males and females DNA on Agarose gel electrophoresis is clearly different. It explains the Tm profiles difference for males and female. 3 independent experiments were performed and show the same result. (TIF) [file pone.0213033.s007.tif]

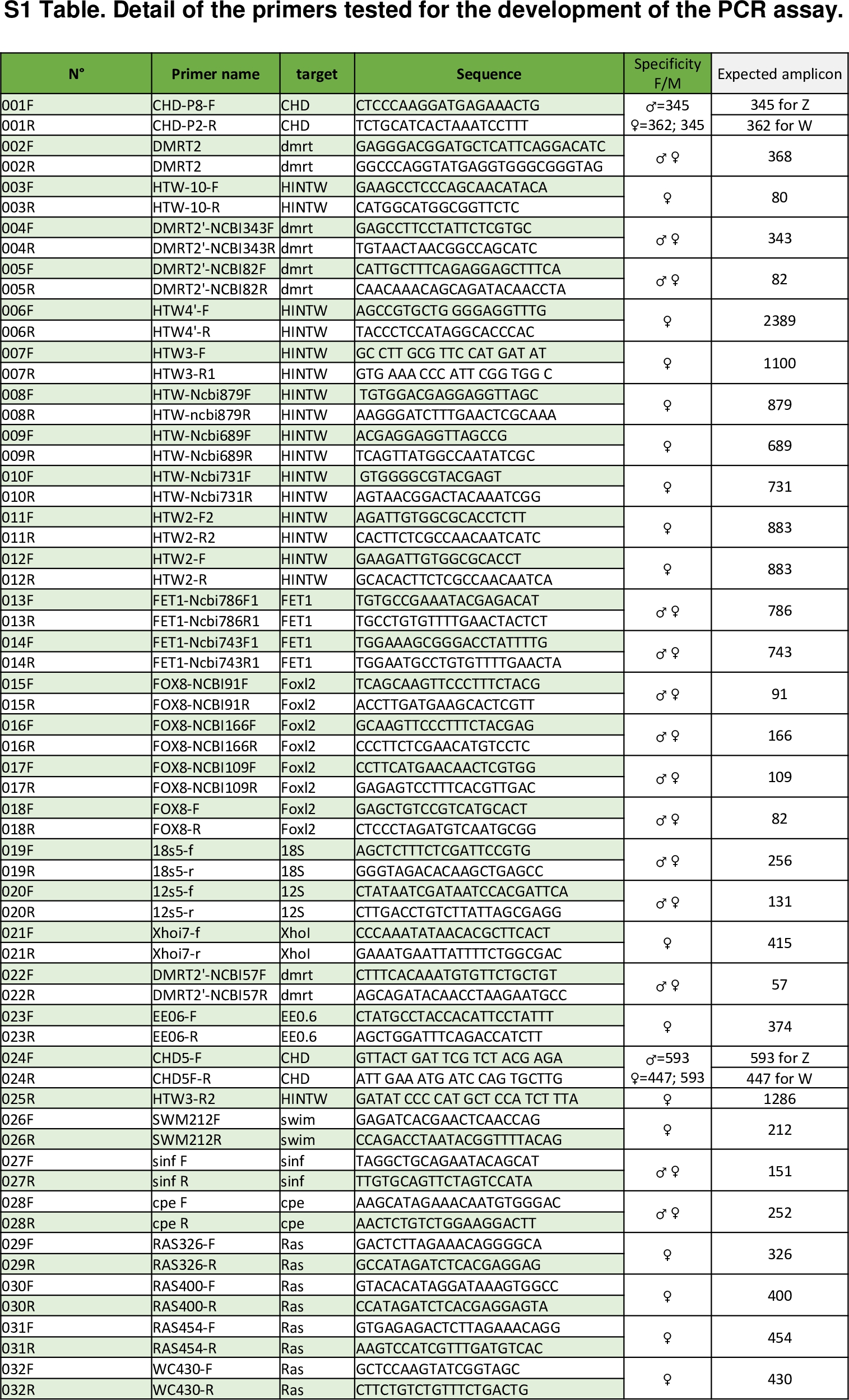

Supplement: S1 Table — (TIF) [file pone.0213033.s008.tif]

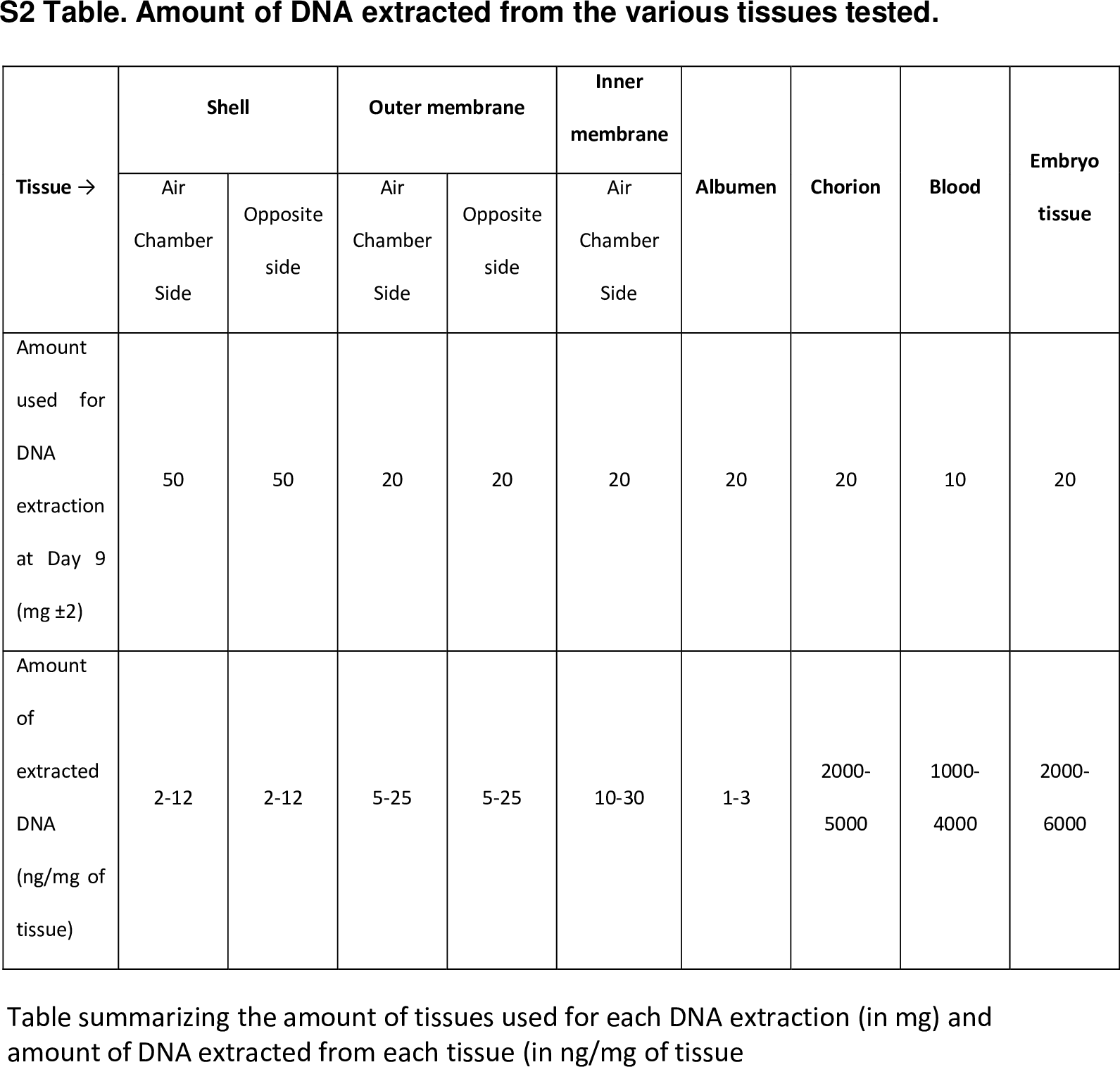

Supplement: S2 Table — Table summarizing the amount of tissues used for each DNA extraction (in mg) and amount of DNA extracted from each tissue (in ng/mg of tissue). (TIF) [file pone.0213033.s009.tif]

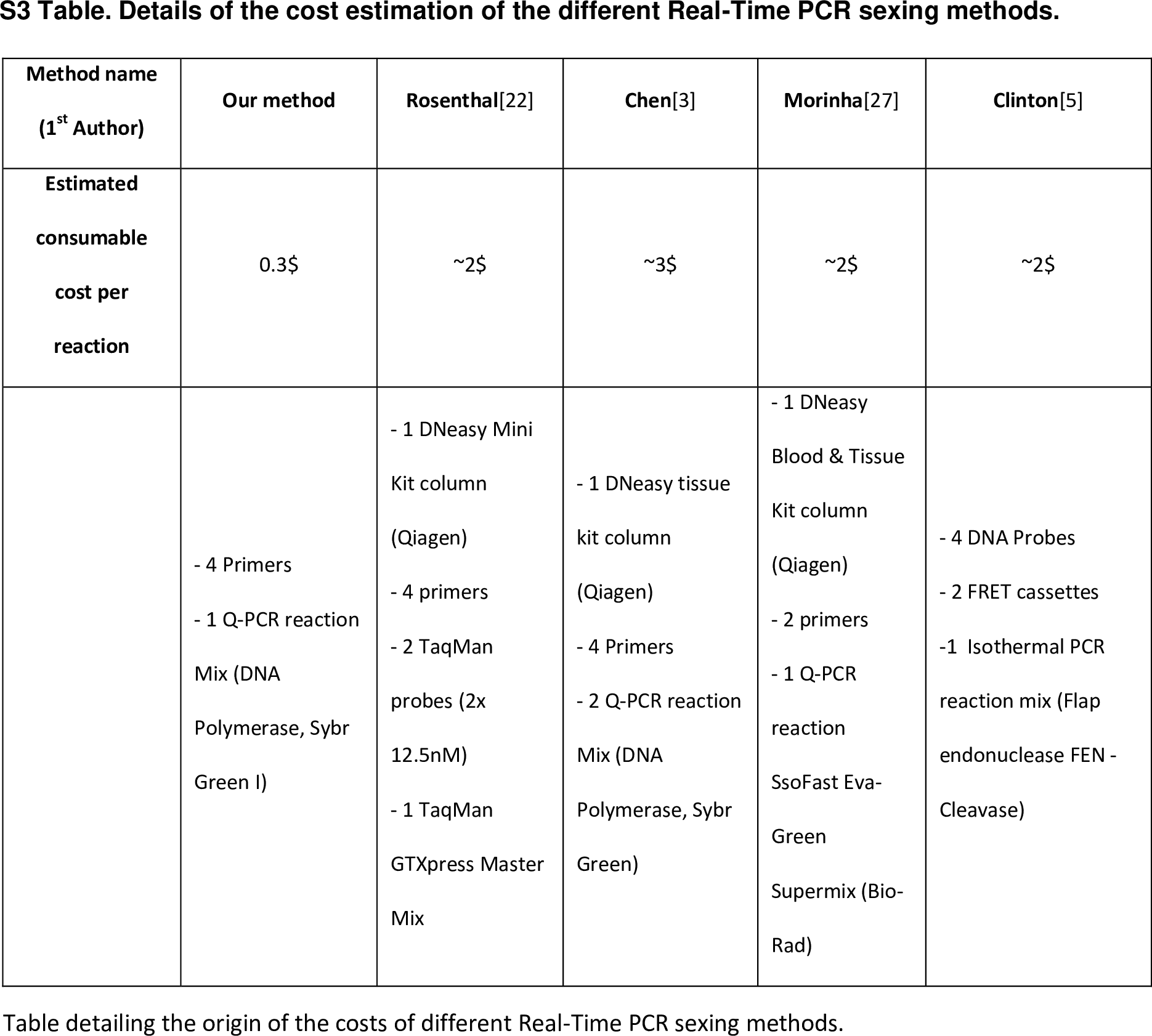

Supplement: S3 Table — (TIF) [file pone.0213033.s010.tif]
